# Consumo de Sal do Himalaia e Sal de Mesa entre Indivíduos Hipertensos

**DOI:** 10.36660/abc.20220243

**Published:** 2022-05-04

**Authors:** Mariana de Souza Dorna, Marcos Mitsuo Seki

**Affiliations:** 1 Departamento de Clínica Médica Universidade Estadual Paulista “Júlio de Mesquita Filho” Botucatu SP Brasil Departamento de Clínica Médica. Universidade Estadual Paulista “Júlio de Mesquita Filho”,Unesp, Botucatu, SP – Brasil

**Keywords:** Hipertensão, Cloreto de Sódio na Dieta, Pressão Arterial, Fatores de Risco, Ingestão de Sal, Urinalise/métodos, Estilo de Vida, Atividade Física

Com acometimento de, aproximadamente, 30% da população brasileira, a hipertensão arterial é elencada com uma das principais causas de Doença Cardiovascular.^1^ A atual recomendação da OMS para ingestão de sódio, é que seja < 2g/pessoa/dia, ou 5g de sal/pessoa/dia. Contudo, já é conhecido que o padrão de ingestão de sal da população brasileira alcança até 12g/dia. Ainda que o aumento pressórico tenha etiologia multifatorial, o consumo excessivo de sódio figura entre as principais causas.^2 , 3^

Esse elemento tem importantes funções fisiológicas como regulação do volume extracelular, condução nervosa e função muscular.^4^ Há pouca divergência na literatura sobre os benefícios da redução do consumo de sódio para população hipertensa. Uma estratégia bastante abordada pelas mídias sociais e impressa foi a adoção do Sal do Himalaia por ser rico em ferro, zinco, cálcio, magnésio e potássio e seus supostos benefícios em detrimento do sal regular no controle pressórico de hipertensos.^4 - 9^ Nesse contexto, Loyola IP et al.^10^ avaliaram o impacto da ingestão de sal de mesa e do sal do Himalaia sobre os parâmetros pressóricos e a concentração urinária de sódio em indivíduos hipertensos.^10^

Nessa publicação, os autores realizaram um estudo randomizado, do tipo crossover em que foram recrutadas mulheres entre 40 e 65 anos. A amostra foi então dividida em 2 grupos: sal do Himalaia e sal de mesa. O período de intervenção foi de 4 semanas para cada tipo de tratamento e, após 2 semanas de washout, houve alternância do tipo de sal por mais 4 semanas. As amostras de sal foram avaliadas e fortificadas com iodo, e foram dadas orientações sobre alimentação e uso dos tipos de sal.^10^

Ao final, 18 mulheres foram consideradas elegíveis para o estudo. A mediana de duração da intervenção foi de 35 dias, e a média de consumo de sal foi de 6,37g e 5,98g de sal do Himalaia e sal de mesa, respectivamente. Apesar da ausência de diferença estatística entre os grupos quanto aos parâmetros pressóricos e concentração urinária de sódio, esse trabalho coloca uma lente de aumento sobre a importância de ensaios clínicos controlados e randomizados sobre a temática. A comercialização e utilização do sal do Himalaia ganhou muita atenção da mídia, em especial pelos supostos efeitos anti-hipertensivos, e esse trabalho fortalece cientificamente a orientação sobre o consumo desse elemento pela população hiper e normotensa. Outro ponto importante a ser ressaltado é o papel fundamental desenvolvido pela mudança de estilo de vida e prática regular de atividade física como estratégias de tratamento para hipertensão arterial.

## References

[1] Malachias M, Souza W, Plavnik F, Rodrigues C, Brandão A, Neves M, et al. 7a Diretriz Brasileira de Hipertensão Arterial (SBC, SBH, SBN, 2016). Arq Bras Cardiol. 2016;107(3):1–103. doi: 10.5935/abc.20160151.

[2] World Health Organization (WHO). Guideline sodium intake for adults and children. (approved by the Guidelines Review Committee). Geneva; 2012. 23658998

[3] Arantes AC, Souza ALL, Vitorino PVO, Jardim PCBC, Jardim TSV, Rezende JM, et al. Efeito da redução de sal de adição sobre a pressão arterial central e periférica. Arq Bras Cardiol. 2020;114(3):554-61. doi: 10.36660/abc.20180426. 10.36660/abc.20180426 PMC7792722 32267330

[4] Moore-Fayet F, Wibisono C, Carr P, Duve E, Petocz P, Lancaster G, et al. An analysis of the mineral composition of Pink Salt available in Australia. Foods.2020; 9(10):1-15. doi: 10.36660/abc.20180426 10.3390/foods9101490 PMC7603209 33086585

[5] Centola D. Social Media and the Science of Health Behavior. Circulation. 2013;127(21):2136-44. doi: 10.1161/CIRCULATIONAHA.112.101816. 10.1161/CIRCULATIONAHA.112.101816 23716382

[6] Aburto NJ, Hanson S, Gutierrez H, Hooper L, Elliott P, Cappuccio FP. Effect of increased potassium intake on cardiovascular risk factors and disease: Systematic review and meta-analyses. BMJ. 2013;346:f1378. doi: 10.1136/bmj.f1378. doi: 10.1136/bmj.f1378. 10.1136/bmj.f1378 PMC4816263 23558164

[7] Kolte D, Vijayaraghavan K, Khera S, Sica DA, Frishman WH. Role of Magnesium in Cardiovascular Diseases. Cardiol Rev. 2014;22(4): 182–92. 10.1097/CRD.0000000000000003 24896250

[8] Livingstone KM, Lovegrove JA, Cockcroft JR, Elwood PC, Janet E, Givens DI, et al. Evidence from the Caerphilly Prospective Study. Hypertension. 2013; 61(1):42-7. doi: 10.1161/HYPERTENSIONAHA.111.00026. 10.1161/HYPERTENSIONAHA.111.00026 23150503

[9] Poirier P, Ph D, Wielgosz A, Ph D, Morrison H, Ph D, et al. Association of urinary sodium and potassium excretion with blood pressure. N Engl J Med. 2014;371(7):601–11. doi: 10.1056/NEJMoa1311989. 10.1056/NEJMoa1311989 25119606

[10] Loyola IP, Sousa MF, Jardim TV, Mendes MM, Barroso WKS, et al. Comparação entre os efeitos da ingestão de sal do Himalaia e de sal comum sobre os valores de sódio urinário e pressão arterial em indivíduos hipertensos. Arq Bras Cardiol. 2022; 118(5):875-882 10.36660/abc.20210069 PMC9368875 35137791

