# Supplementary material for: Consumo de Sal do Himalaia e Sal de Mesa entre Indivíduos Hipertensos
Source: Arq Bras Cardiol. 2022 May 4;118(5):883–4. [Article in Portuguese] doi: 10.36660/abc.20220243 (PMC9368889; doi:10.36660/abc.20220243)
Supplement: Translation

# Translation: Himalayan Salt and Table Salt Intake among Hypertensive Individuals

**Authors:** Mariana de Souza Dorna, Marcos Mitsuo Seki

**Affiliations:** 1 Brazil Departamento de Clínica Médica. Universidade Estadual Paulista “Júlio de Mesquita Filho”, Unesp, Botucatu, SP – Brazil

**Keywords:** Hypertension, Sodium Chloride, Dietary, Risk Factors, Salt Intake, Urinalysis/methods, Life Style, Physical Activity

With the involvement of approximately 30% of the Brazilian population, arterial hypertension is listed as one of the main causes of cardiovascular disease.^1^ The current WHO recommendation for sodium intake is < 2g/person/day, or 5g salt/person/day. However, it is already known that the salt intake pattern of the Brazilian population reaches up to 12g/day. Although the increase in blood pressure has a multifactorial etiology, excessive sodium intake is among the main causes.^2 , 3^

This element has important physiological functions such as regulation of extracellular volume, nerve conduction and muscle function.^4^ There is little disagreement in the literature about the benefits of reducing sodium intake for the hypertensive population. A strategy widely addressed by social media and in print was the adoption of Himalayan Salt for being rich in iron, zinc, calcium, magnesium and potassium and its supposed benefits over table salt in pressure control on hypertensive patients.^4 - 9^ In this context, Loyola IP et al.^10^ evaluated the impact of table salt and Himalayan salt intake on blood pressure parameters and urinary sodium concentration in hypertensive individuals.^10^

In this publication, the authors performed a randomized, crossover study in which women aged between 40 and 65 years were recruited. The sample was then divided into 2 groups: Himalayan salt and table salt. The intervention period was 4 weeks for each type of treatment, and after 2 weeks of washout, there was an alternation of salt type for another 4 weeks. Salt samples were evaluated and fortified with iodine, and guidance was given on diet and the use of types of salt.^10^

In the end, 18 women were considered eligible for the study. The median duration of the intervention was 35 days, and the mean salt intake was 6.37g and 5.98g of Himalayan salt and table salt, respectively. Despite the lack of statistical difference between the groups regarding blood pressure parameters and urinary sodium concentration, this work magnifies the importance of controlled and randomized clinical trials on the subject. The commercialization and use of Himalayan salt have gained a lot of media attention, especially for its supposed antihypertensive effects, and this work scientifically strengthens the orientation on the intake of this element by the hyper and normotensive population. It is important to emphasize the fundamental role of lifestyle changes and regular physical activity as treatment strategies for arterial hypertension.
